# Healthcare utilization and clinical outcomes after ablation of atrial fibrillation in patients with and without insertable cardiac monitoring

**DOI:** 10.1016/j.hroo.2021.12.005

**Published:** 2022-01-07

**Authors:** Moussa C. Mansour, Emily M. Gillen, Audrey Garman, Sarah C. Rosemas, Noreli Franco, Paul D. Ziegler, Jesse M. Pines

**Affiliations:** ∗ Massachusetts General Hospital, Harvard Medical School, Boston, Massachusetts; † Panalgo LLC, Boston, Massachusetts; ‡ Medtronic, Mounds View, Minnesota; § US Acute Care Solutions, Canton, Ohio

**Keywords:** Atrial fibrillation, Ablation, Insertable cardiac monitor, Oral anticoagulation, Healthcare economics

## Abstract

**Background:**

Compared with short-term electrocardiogram (ECG) monitors, insertable cardiac monitors (ICMs) have been shown to increase atrial fibrillation (AF) detection rates and the opportunity to treat recurrent AF in patients postablation.

**Objective:**

To examine healthcare utilization and clinical outcomes following AF ablation, in patients with vs without ICM.

**Methods:**

Retrospective analysis pooling Optum Clinformatics and Medicare Fee-for-service 5% Sample claims databases. Patients with an AF ablation between January 1, 2011, and March 31, 2018 who received an ICM implant within 1 year pre-/postablation were propensity score matched 1:3 to patients without ICM. Outcomes included AF-related healthcare utilization, medication use, and occurrence of composite severe cardiovascular events (stroke / transient ischemic attack, major bleeds, systemic embolism, AF- or heart failure–related hospitalization, or death).

**Results:**

A total of 1000 ICM patients and 2998 non-ICM patients were included. During mean follow-up of 33 ± 16 months postablation, ICM patients experienced significantly fewer severe cardiovascular events (1.09 ± 2.22 vs 1.37 ± 4.19, *P* = .008) and associated costs ($20,757 vs $29,106, *P* = .0005). ICM patients had a greater number of AF-related clinic visits (16.8 vs 11.6 visits, *P* < .0001) and were more likely to receive a repeat ablation (38.7% vs 32.4%, *P* = .0003). Total all-cause costs during follow-up were not statistically different. Discontinuation of oral anticoagulation was higher in ICM patients at 1 year (44% vs 31%, *P* < .0001) and 2 years (73% vs 64%, *P* = .0012).

**Conclusion:**

A shift from acute, reactive care to routine outpatient management was observed in patients with long-term ECG monitoring. Results suggest closer patient management in patients with long-term monitoring after an AF ablation and an improvement in outcomes, at similar overall cost.

**Key Findings**

▪ In a pooled sample of commercially insured and Medicare fee-for-service patients in the United States, 1000 patients receiving insertable cardiac monitor (ICM) within 1 year of ablation for atrial fibrillation (AF) were propensity score matched with 2998 non-ICM patients.
▪ During a mean follow-up of 33 months, ICM patients experienced significantly fewer severe cardiovascular events (1.09 vs 1.37, *P* = .008) and associated costs ($20,757 vs $29,106, *P* = .0005).
▪ A higher frequency of routine clinic-based visits and repeat AF ablations was observed in ICM patients. Overall, a shift in utilization from acute, reactive care to routine outpatient management was observed in patients with long-term electrocardiogram monitoring.
▪ Patients with long-term monitoring were more likely to be discontinued from oral anticoagulants and antiarrhythmics at 1 and 2 years postablation.

## Introduction

In 2017, over 37 million individuals worldwide had prevalent atrial fibrillation (AF) or atrial flutter.^1^ The number of patients with AF is expected to rise partly owing to the aging of the population and an improved ability to diagnose AF through cardiac monitoring and cardiac implantable electronic devices (CIEDs) rather than periodic electrocardiogram (ECG) monitoring, focusing only on when patients are symptomatic. AF is also associated with a poorer quality of life as well as increased risk of stroke and progression to heart failure (HF). In the United States in 2017, the age-adjusted mortality rate from AF was 6.6 per 100,000 people, with higher death rates in both males (OR 1.5, 95% CI = 1.2–1.8]) and females (OR 1.9, 95% CI = 1.5–2.2).^1^^,^^2^ The estimated average per capita medical spending for patients with nonvalvular AF aged 18–64 years was $38,861 (vs $28,506 for matched patients without AF; 2014 US dollars).^3^

Catheter ablation can reduce AF burden, improving symptoms and potentially allowing discontinuation of oral anticoagulation (OAC) medication in some patients. Discontinuation of OAC is desirable where possible, to reduce the risk of adverse events such as bleeding. However, following catheter ablation, symptomatic and asymptomatic AF recurrences are observed in up 70% of patients.^4^, ^5^, ^6^, ^7^, ^8^ Regardless of the presence of symptoms, patients with AF recurrence remain at an increased risk of AF-related thromboembolic events and those at high risk should receive appropriate OAC treatment, according to current AF management guidelines.^9^^,^^10^

For postablation patients in whom discontinuation of OAC is considered, a 2017 consensus statement on AF ablation from HRS/EHRA/ECAS/APHRS/SOLAECE suggested the use of frequent Holter recordings and/or extended ECG cardiac monitoring to assess AF recurrence.^9^ Monitoring with insertable cardiac monitors (ICMs) has been shown to detect more arrhythmia recurrences than short-term ECG monitors. Although a few studies have shown how ICMs can inform medical management of postablation patients (cardioversion, repeat ablations, pacemaker implant, antiarrhythmic drug and OAC management),^11^, ^12^, ^13^, ^14^, ^15^, ^16^ it remains unclear whether ICM monitoring translates into improvements in health outcomes or impacts costs. The aim of the present study was to address this evidence gap by examining healthcare utilization, clinical outcomes, and costs following AF ablation in patients who received an ICM compared to patients without long-term continuous ECG monitoring.

## Methods

### Data source and patient selection

We analyzed data from the Optum® Clinformatics® Commercial and Medicare Advantage patient claims database (January 1, 2007 to March 31, 2019), which includes a total of 63,727,583 patients; and the Medicare Fee-for-service 5% Sample database (January 1, 2010 to December 31, 2018), representing 4,015,934 patients. These nationally representative databases capture de-identified, patient-level health insurance claims data and provide insight into clinical utilization, medical services, and prescription drug records, therefore reflecting real-world patient engagement and treatment patterns. Sample selection and creation of analytic variables were performed using the Instant Health Data platform (Panalgo LLC, Boston, MA). Statistical analyses were undertaken with R, version 3.2.1 (R Foundation for Statistical Computing, Vienna, Austria). Since this was a noninterventional, retrospective, observational study using de-identified data, informed consent was not required from the patient under an institutional review board exemption status. All aspects of this study were conducted in compliance with Health Insurance Portability and Accountability Act of 1996 (HIPAA) regulations and the HIPAA Omnibus Rule of 2013.

**Figure 1:**
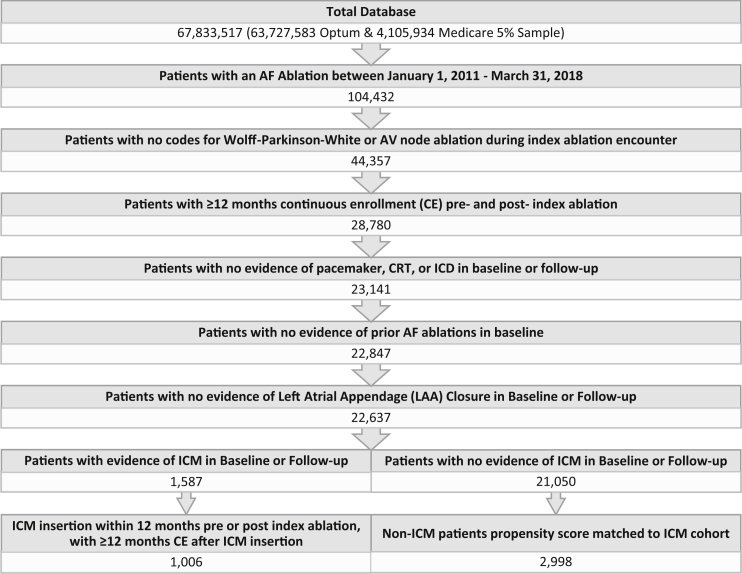
Patient selection flowchart. AF = atrial fibrillation; CE = continuous enrollment; CRT = cardiac resynchronization therapy; ICD = implantable cardioverter-defibrillator; ICM = insertable cardiac monitor; LAA = left atrial appendage.

Patient selection criteria used are summarized in Figure 1. Patients were eligible for the analysis if an ablation procedure (ICD-9 37.34; ICD-10 02583ZZ; CPT 93651, 93655, 93656, 93657) with diagnosis code for AF (ICD-9 427.31; ICD-10 I48.0, I48.1, I48.2, I48.91) during the same encounter took place between January 1, 2011 and March 31, 2018 (index ablation). Patients were required to be continuously enrolled in their health plan for at least 12 months before and 12 months after index ablation (allowing for a gap of up to 32 days in continuous enrollment). The baseline period consisted of the 12 months prior to index ablation, and the follow-up period extended from index ablation date to the end of continuous enrollment or death, whichever came first.

Patients were excluded if they met any of the following criteria: procedure code for AV node ablation (ICD-10 02583ZZ; CTP 93650) or a diagnosis code for Wolff-Parkinson-White syndrome (ICD-9 426.7; ICD-10 I45.6) during index ablation encounter; prior AF ablation; left atrial appendage closure (ICD-10 02L73DK; CPT 33340) during baseline or follow-up periods; CIEDs capable of AF monitoring other than ICMs during baseline or follow-up periods (pacemaker, cardiac resynchronization therapy, or implantable cardioverter-defibrillator) (codes in Supplemental Table 1).

### Patient cohorts

This study used a retrospective matched cohort design, matching 3:1 non-ICM and ICM patients. The ICM cohort was identified based on the presence of a procedure code for ICM device insertion within close proximity to (12 months before to 12 months after) their index ablation procedure (ICD-9-PCS 37.79; ICD-10-PCS 0JH632Z or 0JH602Z; CPT 33282 or 33285) in order to identify patients with ICMs being used for monitoring success of the ablation procedure. These patients were propensity score matched with up to 3 control patients who had no evidence of ICM insertion or ICM monitoring codes at any time in their available claims data during the study period (baseline or follow-up). Because patients with long-term monitoring may differ from patients who are not monitored in ways that may influence downstream healthcare use and costs, the goal of the matching was to reduce confounding by ensuring that cohorts were as similar as possible in their baseline characteristics. Propensity score matching was performed using a caliper width ≤0.02, based on the following 16 variables: age, sex, health plan type (commercial vs Medicare), geographic region, calendar year of index AF ablation (2011–2018), months of follow-up time post Index AF ablation, Charlson comorbidity score, CHA_2_DS_2_-VASc score (exact score), and evidence of the following comorbidities based on presence of a diagnosis code (codes listed in Supplemental Table 2) during baseline in any diagnostic position on the claims: hypertension, HF (including congestive HF), ischemic heart disease, diabetes mellitus, sleep apnea, presence of ≥1 prescription OAC medication during baseline (Y/N), ≥1 prescription rhythm control medication during baseline (Y/N), and total medical and pharmacy healthcare costs during baseline (ICM costs were excluded for purposes of matching).

### Outcome variables

The occurrence of severe cardiovascular events during follow-up was assessed as a composite of the following parameters: AF-related hospitalization, HF-related hospitalization, stroke / transient ischemic attack (TIA), major bleed, systemic embolism, or death. AF- and HF-related hospitalizations were identified based on the presence of an ICD-9/10 diagnosis code for AF or HF in the primary diagnostic position on the inpatient claim, respectively (codes are listed in Supplementary Table 2). Acute stroke/TIA, systemic embolism, and major bleed events were identified based on ICD-9/10 diagnosis codes in the primary position and restricted to emergency department (ED) or hospitalization only. Major bleeds were classified according to clinical guidelines^17^ and included bleeding events seen in the ED or hospitalization involving the brain, gastrointestinal, or other critical organs, or bleeds leading to transfusion or death. AF- and HF-related hospitalizations were also examined independently of the composite outcome.

In addition to severe cardiovascular events, the occurrence of clinically relevant nonmajor (CRNM) bleeds was identified based on the presence of ICD-9/10 diagnosis codes in any position on the claim, and distinguished from major bleeds per the aforementioned guidelines^17^ as bleeds *not* occurring in the defined critical organs, and in the absence of transfusion or death.

Average costs per patient related to clinical events were calculated and represent payer-paid amounts. Additionally, the number of events and total event-related costs were calculated among patients experiencing at least 1 event.

Total all-cause and AF-related healthcare encounters during the follow-up period and their associated expenditures were identified and stratified by site of service, including inpatient, ED, office visits, outpatient hospital, and other (including urgent care, walk-in retail health clinic, home health care hospice, long-term care, skilled nursing facility, or other ambulatory centers not otherwise classified). AF-related healthcare utilization was identified based on the presence of an ICD-9/10 diagnosis code for AF in the primary position on the claim. Recurrent AF ablation procedures, cardioversions, and external ECG monitoring (24-hour through 30-day monitors) during follow-up were also assessed.

AF-related medication usage during follow-up was assessed for 3 medication classes: antiarrhythmic drugs, rate-control medications, and OACs (including both non–vitamin K oral anticoagulants and warfarin). At a cohort level, medication usage over time was measured as the percentage of patients with at least 1 day’s worth of filled prescription during each 3-month period, from the 3 months prior to index ablation through 2 years of follow-up. Additionally, for the patients taking each class of medication at index, discontinuation (defined as a gap of ≥90 days in medication prescription) was calculated at 1 and 2 years of follow-up. Discontinuation of OACs was assessed in patients with CHA_2_DS_2_-VASc scores ≥2. Medication usage was assessed in the Optum database only, as those data are not fully represented in the Medicare 5% database.

### Statistical analyses

The Pearson χ^2^ test was used to calculate the *P* values for categorical variables. Continuous variables were evaluated using an unpaired *t* test. Where the sample size was small, the Fisher exact test was used.

## Results

### Study cohort

After patient selection according to the identification criteria outlined in Figure 1, a total of 1006 ICM patients were identified (695 from the Optum database; 311 from the Medicare 5% Sample), as well as 21,050 non-ICM patients (15,099 Optum and 5951 Medicare 5%). The propensity score matching process was applied separately in each database and resulted in a final pooled sample of 1000 ICM patients (691 Optum and 309 Medicare 5%) and 2998 non-ICM patients (2073 Optum and 925 Medicare 5%). Patients were excluded if a closely matching patient could not be found in the opposite cohort; ultimately 6 ICM patients and 18,052 non-ICM patients were excluded during the matching process. Baseline characteristics before and after propensity score matching are shown for each database in Table 1. All patient characteristics were balanced after the matching process (Table 2). Mean age was approximately 66.5 years, 40% were female, and the mean ± SD CHA_2_DS_2_-VASc score was 2.6 ± 1.5. Approximately 21% of patients had a history of HF. The mean follow-up duration after index ablation was 33 ± 16 months (median 29 months). Among ICM patients, 39.4% had received the ICM in the 12 months prior to index AF ablation, at a median (interquartile range) of 117 (53–197) days before index, while 60.6% received the ICM during or after the index ablation, at a median (interquartile range) of 43 (1–151) days after ablation date.

**Table 1:** Patient characteristics before and after propensity score matching

Optum database	Before matching			After matching		
	ICM (N = 695)	Non-ICM (N = 15,099)	*P* value	ICM (N = 691)	Non-ICM (N = 2073)	*P* value
Age (years), mean	64.6	64.4	.4481	64.6	65.0	.1203
Female, %	37.2%	33.2%	.0003	37.2%	37.8%	.6544
Medicare Advantage	49.8%	51.2%	.2098	49.8%	50.8%	.3348
Region, n (%)			.9001			.9429
Midwest	182 (26.3%)	3937 (26.2%)		181 (26.2%)	540 (26.1%)	
Northeast	65 (9.4%)	1322 (8.8%)		65 (9.4%)	194 (9.4%)	
South	287 (41.5%)	6179 (41.1%)		287 (41.5%)	885 (42.7%)	
West	158 (22.8%)	3586 (23.9%)		158 (22.9%)	454 (21.9%)	
CHAD_2_S_2_-VASc, mean (SD)†	2.29 (1.53)	2.17 (1.51)	.0374	2.29 (1.53)	2.29 (1.53)	1.0000
CHAD_2_S_2_-VASc, median (range)	2 (1–3)	2 (1–3)		2 (1–3)	2 (1–3)	
Charlson score	1.39	1.40	.7748	1.39	1.41	.6738
Total healthcare costs in baseline (excluding ICM cost)	$29,602	$27,492	.0101	$29,263	$29,661	.7413
Patient history, %						
Heart failure	20.0%	21.3%	.1624	20.0%	21.4%	.2369
Hypertension	79.0%	75.9%	.0012	79.0%	79.4%	.7466
Ischemic heart disease	41.1%	39.6%	.1842	41.1%	42.4%	.3935
Diabetes	23.2%	24.2%	.3189	23.2%	24.2%	.4084
Sleep apnea	34.2%	26.6%	<.0001	34.2%	32.9%	.3148
Oral anticoagulant use	73.6%	70.1%	.0006	73.7%	73.8%	.9151
Antiarrhythmic use	74.0%	70.9%	.0031	74.0%	73.6%	.7749
Year of index ablation, %						
2012	2.8%	13.2%	<.0001	2.8%	2.6%	.5863
2013	4.2%	10.6%	<.0001	4.2%	3.9%	.4309
2014	11.7%	11.4%	.6845	11.7%	12.0%	.7829
2015	17.7%	13.6%	<.0001	17.7%	18.6%	.4201
2016	24.2%	14.4%	<.0001	24.2%	22.6%	.1681
2017	28.4%	19.5%	<.0001	28.4%	29.1%	.5708
2018	8.7%	6.3%	.0002	8.7%	8.9%	.7829

Medicare 5% database	ICM (N = 311)	Non-ICM (N = 5951)	*P* value	ICM (N = 309)	Non-ICM (N = 925)	*P* value‡
Age (years), mean	70.4	70.4	.8396	70.4	70.7	.2901
Female, %	45.0%	41.8%	.0623	45.0%	48.0%	.2004
Medicare FFS	100.0%	100.0%	1.000	100.0%	100.0%	1.000
Region, n (%)			.0388			.9176
Midwest	45 (14.5%)	1243 (20.9%)		45 (14.6%)	131 (14.2%)	
Northeast	46 (14.8%)	910 (15.3%)		46 (14.9%)	133 (14.4%)	
South	149 (48.1%)	2653 (44.6%)		149 (48.2%)	467 (50.5%)	
West	70 (22.6%)	1143 (19.2%)		69 (22.3%)	194 (21.0%)	
CHA_2_DS_2_-VASc, mean (SD)†	3.17 (1.36)	3.07 (1.39)	.2283	3.17 (1.36)	3.17 (1.36)	.9967
CHA_2_DS_2_-VASc, median (range)	3 (2–4)	3 (2–4)		3 (2–4)	3 (2–4)	
Charlson score	1.71	1.90	.0028	1.71	1.70	.9096
Total healthcare costs in baseline (excluding ICM cost)	$14,972	$14,637	.6140	$14,975	$14,467	.5158
Patient history, %						
Heart failure	20.4%	28.4%	.0000	20.4%	22.0%	.4358
Hypertension	88.0%	86.5%	.1915	88.0%	90.2%	.1476
Ischemic heart disease	50.7%	52.3%	.3418	50.7%	50.3%	.6330
Diabetes	31.3%	32.3%	.5395	31.4%	36.0%	.0324∗
Sleep apnea	30.4%	24.7%	.0005	30.3%	28.0%	.3038
Oral anticoagulant use	9.7%	5.1%	<.0001	9.6%	9.5%	.5873
Antiarrhythmic use	17.0%	8.6%	<.0001	16.7%	16.5%	.755
Year of index ablation, %						
2012	2.6%	13.1%	<.0001	2.6%	2.2%	.2254
2013	2.6%	12.4%	<.0001	2.6%	3.1%	.2755
2014	13.9%	13.3%	.6129	14.0%	14.4%	.9394
2015	22.3%	15.8%	<.0001	22.3%	22.8%	.6422
2016	29.5%	15.6%	<.0001	29.4%	27.1%	.2220
2017	25.9%	18.4%	<.0001	26.0%	27.6%	.4071
2018	-	-	-	-	-	-

ICM = insertable cardiac monitor.

† Patients were matched on exact CHA_2_DS_2_-VASc score.

‡ Statistically significant difference between the 2 cohorts is indicated by an asterisk.

**Table 2:** Patient characteristics of final pooled sample (Optum and Medicare 5% Fee-for-service)

Variable	ICM (N = 1000)	Non-ICM (N = 2998)	*P* value
Age (years), mean ± SD	66.38 ± 9.28	66.71 ± 9.47	.3222
Female, n (%)	396 (39.60%)	1227 (40.93%)	.4821
Medicare Fee-for-service & Medicare Advantage, n (%)	653 (65.30%)	1977 (65.94%)	.739
Region, n (%)			.8518
Midwest	226 (22.60%)	687 (22.92%)	
Northeast	111 (11.10%)	323 (10.77%)	
South	436 (43.60%)	1342 (44.76%)	
West	227 (22.70%)	646 (21.55%)	
CHA_2_DS_2_-VASc, mean ± SD†	2.56 ± 1.53	2.56 ± 1.53	.9958
CHA_2_DS_2_-VASc, median	2 (1–4)	2 (1–4)	
Charlson score	1.49 ± 1.66	1.50 ± 1.77	.8629
Patient history, n (%)			
Heart failure	201 (20.10%)	2474 (82.52%)	.5525
Hypertension	818 (81.80%)	2474 (82.52%)	.6381
Ischemic heart disease	441 (44.10%)	1340 (44.70%)	.7704
Diabetes	257 (25.70%)	823 (27.45%)	.2987
Sleep apnea	330 (33.00%)	951 (31.72%)	.4769
Oral anticoagulant use	539 (53.90%)	1622 (54.10%)	.9404
Antiarrhythmic use	563 (56.30%)	1677 (55.94%)	.8703
Year of index ablation			.9653
2011	27 (2.70%)	75 (2.50%)	
2012	27 (2.70%)	71 (2.37%)	
2013	37 (3.70%)	110 (3.67%)	
2014	124 (12.40%)	387 (12.91%)	
2015	191 (19.10%)	606 (20.21%)	
2016	258 (25.80%)	726 (24.22%)	
2017	276 (27.60%)	841 (28.05%)	
2018	60 (6.00%)	182 (6.07%)	

ICM = insertable cardiac monitor.

† Patients were matched on exact CHA_2_DS_2_-VASc score.

### Acute clinical events and costs during follow-up

**Figure 2:**
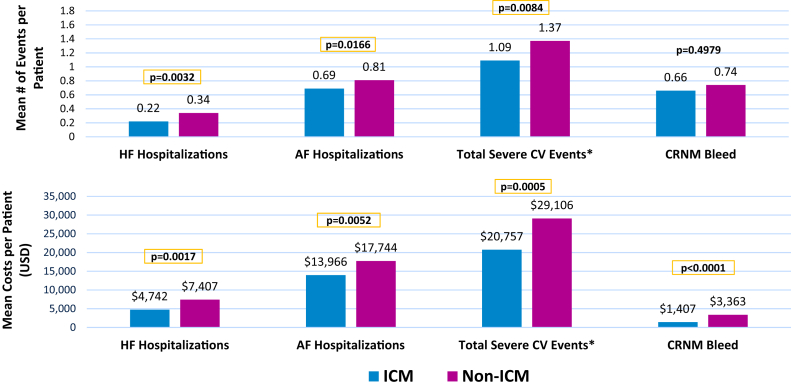
Clinical events and associated expenditures during follow-up. ∗Composite of: acute ischemic stroke, transient ischemic attack, systemic embolism, major bleeds, AF- and HF-related hospitalizations, and death. AF = atrial fibrillation; CRNM = clinically relevant nonmajor bleed; CV = cardiovascular; HF = heart failure.

The occurrence of and costs related to severe cardiovascular clinical events are shown in Figure 2. During the average 33 months of follow-up, the composite outcome of severe cardiovascular events was experienced by nonsignificantly fewer ICM patients (39.7% vs 41.8%, *P* = .2516); however, the average number of events was significantly lower: mean 1.09 ± 2.22 events vs 1.37 ± 4.19 events, *P* = .008. Total per-patient costs related to severe cardiovascular events were on average $8349 lower in ICM patients ($20,757 ± $49,426 vs $29,106 ± $99,272, *P* = .0005). In patients with events, total event-related costs per patient averaged $52,285 in ICM patients compared with $69,586 in non-ICM patients (*P* = .0011). This reduction in total event-related costs appears to be driven primarily by the lower frequency of events (2.75 vs 3.27, respectively, *P* = .00194) in these patients, as the cost per event was not different between ICM and non-ICM patients (Table 3).

**Table 3:** Clinical events during follow-up post index ablation

Clinical events	ICM (N = 1000)	Non-ICM (N = 2998)	*P* value†
Severe CV events,‡ n (%)	397 (39.70%)	1254 (41.83%)	.2516
No. of events per patient, mean ± SD	1.09 ± 2.22	1.37 ± 4.19	.0084∗
Total event-related costs per patient	$20,757 ± $49,426	$29,106 ± $99,272	.0005∗
No. of events in patients with ≥1 event	2.75 ± 2.80	3.27 ± 5.98	.0194∗
Total event-related costs in patients with ≥1 event	$52,285 ± $67,158	$69,586 ± $144,058	.0011∗
Cost per event	$20,437 ± $20,178	$22,047 ± $49,646	.3523
AF hospitalizations, n (%)	371 (37.10%)	1184 (39.49%)	.1913
No. of events per patient, mean ± SD	0.69 ± 1.24	0.81 ± 2.03	.0166∗
Total event-related costs per patient	$13,966 ± $30,539	$17,744 ± $51,823	.0052∗
No. of events in patients with ≥1 event	1.85 ± 1.41	2.06 ± 2.81	.0496∗
Total event-related costs in patients with ≥1 event	$37,645 ± $40,303	$44,931 ± $74,707	.0158∗
Cost per event	$22,013 ± $21,708	$23,652 ± $51,046	.3792
HF hospitalizations, n (%)	126 (12.60%)	441 (14.71%)	.1088
No. of events per patient, mean ± SD	0.22 ± 0.78	0.34 ± 1.85	.0032∗
Total event-related costs per patient	$4742 ± $19,067	$7407 ± $32,507	.0017∗
No. of events in patients with ≥1 event	1.71 ± 1.50	2.31 ± 4.34	.0165∗
Total event-related costs in patients with ≥1 event	$37,633 ± $40,717	$50,351 ± $70,925	.0107∗
Cost per event	$23,604 ± $27,367	$26,388 ± $35,243	.3478
CRNM bleeds, n (%)	141 (20.41%)	412 (19.87%)	.8049
No. of events per patient, mean ± SD	0.50 ± 1.56	0.69 ± 6.48	.2117
Total event-related costs per patient	$1407 ± $6580	$3528 ± $25,203	<.0001∗
No. of events in patients with ≥1 event	2.79 ± 3.43	3.34 ± 11.36	.2668
Total event-related costs in patients with ≥1 event	$5964 ± $12,521	$15,929 ± $51,706	<.0001∗
Cost per event	$3699 ± $9866	$8229 ± $27,820	.0003^∗^

AF = atrial fibrillation; CRNM = clinically relevant nonmajor; CV = cardiovascular; HF = heart failure; ICM = insertable cardiac monitor.

† Statistically significant differences between the 2 cohorts are indicated by an asterisk.

‡ Severe CV events represent a composite of AF-related hospitalization, HF-related hospitalization, stroke / transient ischemic attack, major bleed, systemic embolism, or death.

The mean numbers of AF and HF hospitalizations were significantly lower in ICM patients: 0.69 ± 1.24 vs 0.81 ± 2.03 AF-related hospitalizations (*P* = .0166) and 0.22 ± 0.78 vs 0.34 ± 1.85 HF-related hospitalizations (*P* = .0032). Correspondingly, average total per-patient costs related to AF- and HF-related hospitalizations were lower in the ICM cohort, while the cost per event was nondifferent between cohorts (Table 3).

Total costs associated with CRNM bleeds were also lower in ICM patients compared to the matched controls ($1407 vs $3528, *P* < .0001); however, unlike the other clinical event types, this was driven by a lower cost per event in ICM patients vs non-ICM, while the average number of events per patient was nonsignificantly lower with ICM (0.50 vs 0.69, *P* = .2117).

### Procedures during follow-up

ICM patients were more likely to undergo a repeat AF ablation during follow-up: 387 (38.7%) vs 972 (32.4%) patients, *P* = .0003, with a mean of 0.54 ± 0.86 vs 0.40 ± 0.68 ablations per patient, *P* < .0001. Conversely the rate of electrical cardioversion was lower in the ICM cohort: 217 (21.7%) vs 805 (26.9%) patients, *P* = .0014.

Fewer than half of the non-ICM patients (45.8%, 1374 patients) received an external ambulatory ECG monitor during follow-up, inclusive of Holter monitors, extended Holter monitors, or 30-day monitoring with an external loop recorder or mobile cardiac outpatient telemetry.

## Total healthcare utilization at baseline and follow-up

Table 4 shows total all-cause and AF-related healthcare utilization by site of service during the baseline period (12 months prior to index ablation) and the follow-up period (from index ablation to end of continuous enrollment or death). Associated expenditures are also reported, including any costs related to ICM device, placement, and monitoring.

**Table 4:** All-cause and atrial fibrillation–related healthcare utilization by site of service

	Baseline			Follow-up		
	ICM (N = 1000)	Non-ICM (N = 2998)	*P* value†	ICM (N = 1000)	Non-ICM (N = 2998)	*P* value†
All-cause healthcare utilization						
Total, n (%)	1000 (100%)	2997 (99.97%)	1	1000 (100%)	2998 (100%)	1
No. of encounters	58.15 ± 40.38	57.18 ± 41.27	.5123	157.34 ± 122.95	147.15 ± 129.36	.0252∗
Costs per patient	$24,194 ± $26,437	$20,594 ± $36,792	.0008∗	$91,460 ± $78,635	$85,996 ± $104,524	.0815
Inpatient	352 (35.20%)	973 (32.45%)	.1192	396 (39.60%)	1238 (41.29%)	.3646
No. of encounters	0.49 ± 0.83	0.47 ± 0.99	.7382	0.79 ± 1.47	0.92 ± 2.38	.0396∗
Costs per patient	$6706 ± $15,602	$6977 ± $22,909	.6755	$15,451 ± $32,692	$19,405 ± $54,179	.0058∗
ED	429 (42.90%)	1297 (43.26%)	.8702	548 (54.80%)	1574 (52.50%)	.2207
No. of encounters	0.82 ± 1.36	0.83 ± 1.49	.8409	1.65 ± 3.41	1.47 ± 3.93	.1858
Costs per patient	$2352 ± $5482	$2360 ± $5999	.9663	$4563 ± $11,985	$4397 ± $23,754	.7737
Outpatient hospital	889 (88.90%)	2594 (86.52%)	.0591	942 (94.20%)	2804 (93.53%)	.4959
No. of encounters	6.95 ± 9.79	6.49 ± 10.02	.2035	17.51 ± 22.84	16.62 ± 31.89	.3392
Costs per patient	$9210 ± $16,556	$6080 ± $17,944	<.0001∗	$42,936 ± $53,832	$38,905 ± $60,844	.0475∗
Office	951 (95.10%)	2831 (94.43%)	.4673	953 (95.30%)	2861 (95.43%)	.8618
No. of encounters	17.28 ± 13.62	16.41 ± 12.88	.0777	49.19 ± 41.51	40.92 ± 39.72	<.0001∗
Costs per patient	$3070 ± $3396	$2794 ± $3665	.0295∗	$7346 ± $8354	$6566 ± $8434	.0108∗
Other‡	947 (94.70%)	2769 (92.36%)	.0151^∗^	986 (98.60%)	2929 (97.70%)	.1088
No. of encounters	32.63 ± 34.80	32.98 ± 35.79	.7832	88.21 ± 104.25	87.21 ± 105.19	.7934
Costs per patient	$2856 ± $6040	$2382 ± $9450	.0659	$21,163 ± $39,518	$16,723 ± $30,659	.0012∗
AF-related healthcare utilization						
Total, n (%)	976 (97.60%)	2899 (96.70%)	.1852	1000 (100%)	2998 (100%)	1
No. of encounters	11.66 ± 8.29	11.43 ± 8.98	.4584	31.17 ± 22.74	23.64 ± 24.83	<.0001∗
Costs per patient	$15,438 ± $18,599	$13,018 ± $25,335	.0012∗	$71,029 ± $56,391	$65,861 ± $77,234	.0231∗
Inpatient	322 (32.20%)	905 (30.19%)	.2478	371 (37.10%)	1184 (39.49%)	.1913
No. of encounters	0.42 ± 0.71	0.42 ± 0.85	.9337	0.69 ± 1.24	0.81 ± 2.03	.0166∗
Costs per patient	$5767 ± $13,648	$6150 ± $21,154	.5080	$13,966 ± $30,539	$17,744 ± $51,823	.0052∗
ED	308 (30.80%)	968 (32.29%)	.4037	381 (38.10%)	1,073 (35.79%)	.2017
No. of encounters	0.47 ± 0.92	0.53 ± 1.07	.1228	0.85 ± 2	0.77 ± 2.84	.3352
Costs per patient	$1626 ± $4720	$1728 ± $5029	.5615	$3080 ± $9261	$3001 ± $20,892	.8699
Outpatient hospital	792 (79.20%)	2274 (75.85%)	.0335∗	914 (91.40%)	2666 (88.93%)	.0312^∗^
No. of encounters	3.46 ± 4.99	3.19 ± 4.96	.1379	8.09 ± 11.23	6.63 ± 11.30	.0004∗
Costs per patient	$5811 ± $9004	$3249 ± $6499	<.0001∗	$36,166 ± $35,978	$31,249 ± $39,432	.0003∗
Office	868 (86.80%)	2572 (85.79%)	.4563	907 (90.70%)	2686 (89.59%)	.3451
No. of encounters	5.88 ± 5.37	5.80 ± 5.74	.6782	16.75 ± 15.33	11.56 ± 13.63	<.0001∗
Costs per patient	$989 ± $1184	$974 ± $2321	.7957	$2143 ± $2127	$1656 ± $2069	<.0001∗
Other‡	378 (37.80%)	1,098 (36.62%)	.5292	641 (64.10%)	1,835 (61.21%)	.1110
# of Encounters	1.42 ± 4.19	1.49 ± 4.17	.4194	4.80 ± 12.63	3.87 ± 13.74	.0468∗
Costs per patient	$1,245 ± $4,084	$904 ± $3,766	.0199∗	$15,674 ± $34,017	$12,212 ± $26,123	.0033∗

Data represent n (%) of patients or mean ± standard deviation.

AF = atrial fibrillation; ED = emergency department; ICM = insertable cardiac monitor.

Note: Cohorts were matched on total costs in baseline minus any ICM-related costs. However, ICM-related costs are included above, hence the difference in total costs during baseline.

† Statistically significant differences between the 2 cohorts are indicated by an asterisk.

‡ Other sites of service include urgent care, walk-in retail health clinic, home health care hospice, long-term care, skilled nursing facility, or other ambulatory centers not otherwise classified.

During baseline preablation, all-cause and AF-related healthcare utilization was similar between the patient cohorts, with the exception of higher outpatient hospital costs in the ICM cohort, driven by the ICM insertion procedure. It should be noted that patients had been matched on total healthcare costs during the baseline period as part of the propensity score matching process.

In the follow-up period, a shift was observed in the healthcare utilization between cohorts, as ICM patients had fewer AF-related inpatient hospitalizations and associated costs, but higher physician office and outpatient hospital utilization, compared to non-ICM patients. The number of AF-related physician office visits was 16.8, vs 11.6 in the ICM and non-ICM patients, respectively (*P* < .0001). The higher number of AF-related outpatient hospital encounters in ICM patients (8.1 vs 6.6, *P* = .0004) was driven in part by ICM-related insertion and removal procedures and a greater rate of repeat AF ablations.

All-cause hospitalization rate in ICM patients was lower, attributable largely to the significant reduction in AF-related hospitalizations noted above. Among patients with hospitalizations, the average length of stay was 4.8 ± 3.6 days in ICM vs 5.4 ± 5.5 days in non-ICM patients (*P* = .0250).

Total all-cause healthcare costs were not statistically different between groups ($91,460 ICM vs $85,996 non-ICM, *P* = .0815).

### Usage of AF-related medications

**Figure 3:**
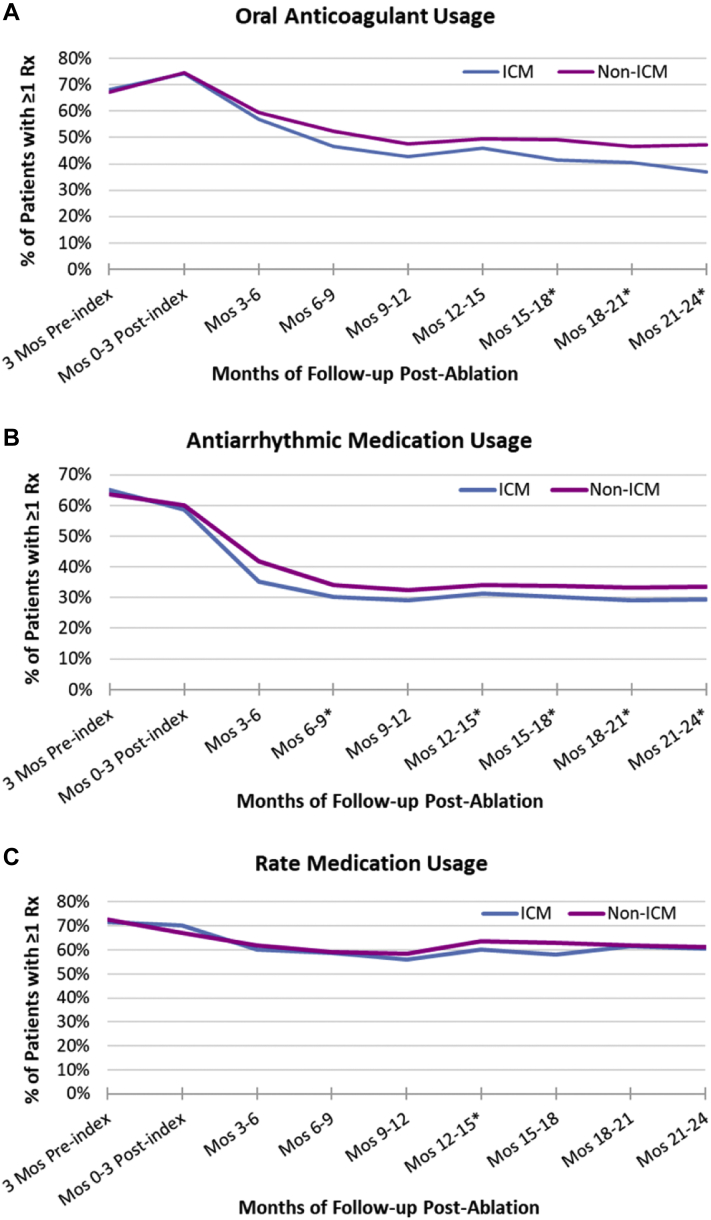
Medication usage during follow-up. **A:** Oral anticoagulant usage. **B:** Antiarrhythmic medication usage. **C:** Rate medication usage. Data indicate the percentage of patients with at least 1 day of prescription coverage for the medication during the 3-month period. Oral anticoagulants include both non–vitamin K antagonist oral anticoagulants and warfarin. ∗Statistically significant difference between the 2 cohorts. ICM = insertable cardiac monitor.

Figure 3 displays the proportion of Optum patients with at least 1 prescription for OAC, antiarrhythmic, and rate control medications, respectively, during each 3-month interval of follow-up. Use of OAC and antiarrhythmics decreased over follow-up in both cohorts. During the first 3 months postablation approximately three-quarters (74%–75%) of patients had evidence of OAC use, whereas at 2 years of follow-up only 37% of ICM patients and 47% of non-ICM patients had evidence of OAC use. Usage of OACs decreased more quickly in ICM patients compared to non-ICM patients, with the proportion of patients on OACs becoming statistically significantly lower at month 15 of follow-up. Use of antiarrhythmic drugs also decreased markedly over follow-up, from 59%–60% to 29%–34%, with a lower usage rate observed in ICM patients starting at month 6. Use of rate control medication was relatively steady during follow-up, decreasing only slightly, from 67%–70% to 60%–61%, and was very similar between cohorts.

**Figure 4:**
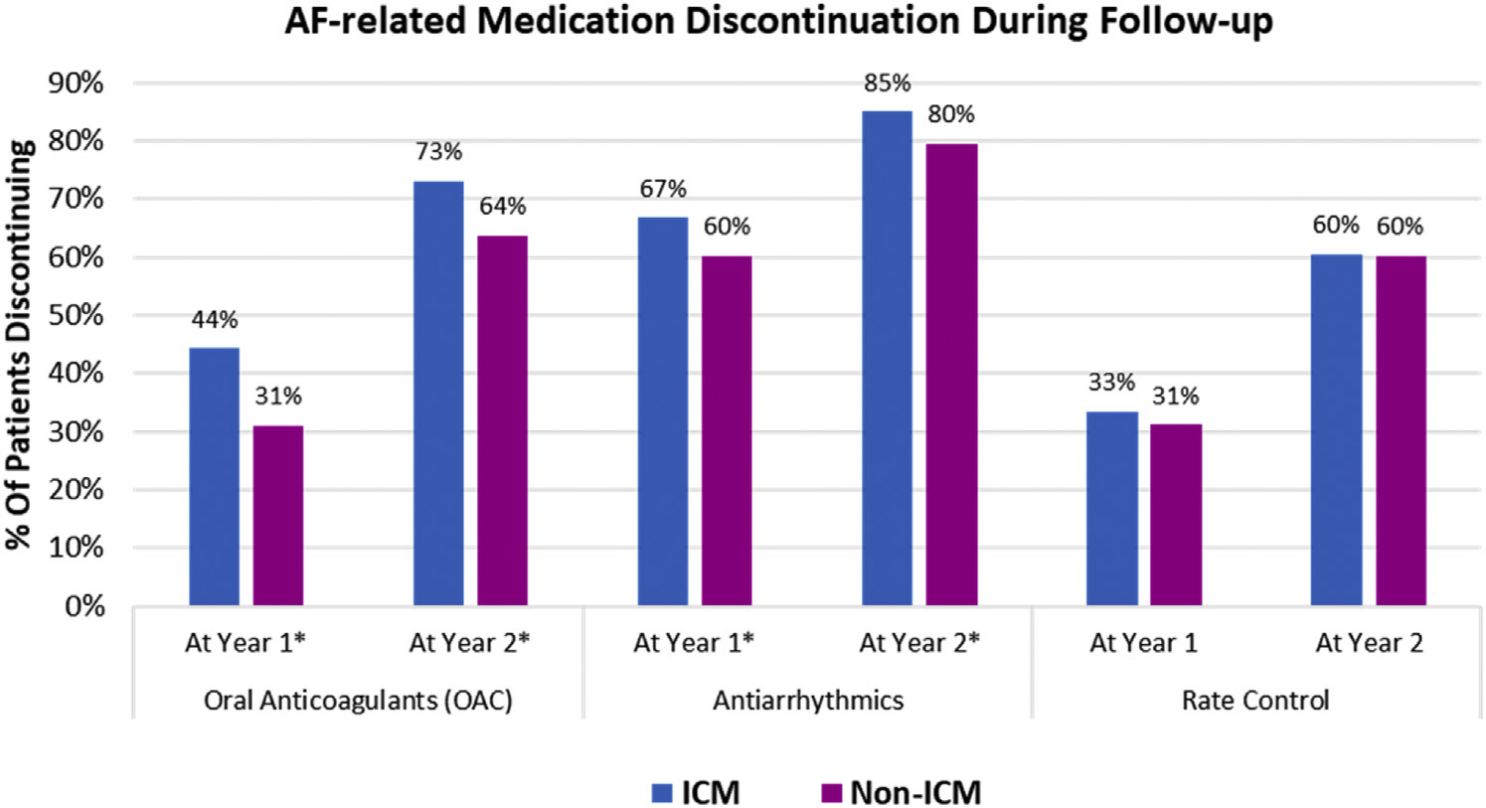
Medication discontinuation during follow-up. Oral anticoagulant (OAC) discontinuation as shown was assessed only in patients with CHA_2_DS_2_-VASc score ≥2. When including patients with CHA_2_DS_2_-VASc = 1, the discontinuation rates were 50% of insertable cardiac monitor (ICM) patients vs 39% of non-ICM patients at year 1 (*P* < .001) and 77% vs 69%, respectively, at year 2 (*P* = .001). In patients with CHA_2_DS_2_-VASc = 1 alone (n = 165 ICM patients and n = 471 non-ICM patients), discontinuation rates were 63% of ICM patients and 60% of non-ICM patients at year 1 (*P* = .085) and 85% vs 82%, respectively, at year 2 (*P* = .408). ∗Statistically significant difference between the 2 cohorts. AF = atrial fibrillation.

At a patient level, medication discontinuation (defined as a gap in coverage ≥90 days) was assessed at 1 year and 2 years of follow-up (Figure 4). A greater proportion of ICM patients had discontinued OACs at 1 year (44% vs 31%, *P* < .0001) and 2 years (73% vs 64%, *P* = .0012) of follow-up. Discontinuation of antiarrhythmic drugs was slightly higher in the monitored population at 1 year (44% vs 31%, *P* < .0001) and 2 years (73% vs 64%, *P* = .0012), whereas there was no difference between cohorts in the discontinuation of rate control medications.

## Discussion

This study examined healthcare utilization/costs, AF-related medication usage, and acute cardiovascular events in patients with and without long-term cardiac monitoring with ICM after an AF ablation. Overall, a shift in utilization from acute, reactive care to routine outpatient management was observed in patients with long-term monitoring. Fewer hospitalizations and severe acute cardiovascular events were experienced in the ICM cohort. Conversely, a greater number of AF-related clinic and outpatient hospital visits (including repeat AF ablation procedures) were observed in ICM patients, which may indicate closer management of these patients and may have contributed to the observed reduction in hospitalizations. For example, the increased rate of repeat AF ablations we observed may have been driven by the greater ability to detect asymptomatic AF and treat subclinical AF recurrence in patients with continuous monitoring.

Indeed, studies have shown an AF recurrence rate of up to 70% of patients during long-term follow-up after successful ablation,^18^, ^19^, ^20^ with the majority of recurrences being asymptomatic in nature.^8^^,^^21^ Although recurrences after ablation are common, the overall AF burden is generally reduced after the initial ablation,^22^^,^^23^ such that intermittent monitoring is less likely than continuous long-term monitoring to capture recurrences or reliably quantify AF burden; this is particularly important for the few patients who have occasional long episodes that could put them at increased risk of stroke and cardiovascular complications.^23^^,^^24^ Likewise, device-based classification of AF has been shown to enhance clinicians’ ability to classify AF burden compared to relying on clinical assessment alone.^25^ Furthermore, we found that fewer than half of the non-ICM patients had any short-term monitoring, placing this group at higher risk for the nondetection and nontreatment of recurrent AF.

Additionally, it was observed that patients with long-term monitoring were more likely to be discontinued from OAC and antiarrhythmic medications postablation. While discontinuation of AF medications (in particular anticoagulants) is a common desire among patients following an ablation procedure, continuous monitoring for AF is recommended in the context of discontinuation in patients with apparently resolved AF.^9^ Several studies have demonstrated successful use of ICM monitoring to inform AF burden–guided OAC management^13^^,^^16^^,^^26^ and antiarrhythmic therapy,^15^ as well as guiding reablation in appropriate patients.^27^^,^^28^

Although a shift in healthcare utilization was observed, the average overall total per-patient healthcare costs were not significantly different, as the increase in routine patient visits and therapeutic interventions in the ICM cohort offset the reduction in acute event-related costs. Interestingly, when examining acute events, while the proportion of patients who experienced an event was similar between the 2 cohorts, ICM patients experienced less frequent events, leading to the lower total acute event-related costs per patient. Additionally, acute events incurred a shorter average length of stay and a lower rate of cardioversions in the ICM cohort. These results may be a result of closer management of ICM patients and the ability to intervene earlier upon AF recurrence. Although the rate of CRNM bleeds was not significantly lower in the ICM cohort, when bleeds occurred they appeared to be less severe (ie, less costly per event), which could be a result of discontinuation of OAC of higher-risk patients in the non-ICM cohort.

These results suggest that long-term cardiac monitoring with ICM is associated with closer management of AF and a lower rate of acute healthcare utilization, at similar total costs from a payer perspective. The findings may support future randomized trials to compare ICM vs other strategies upon AF ablation. Prior to propensity score matching, we observed that ablated patients who receive ICM were more likely to be female, with higher CHA_2_DS_2_-VASc score, higher baseline healthcare costs, underlying hypertension and sleep apnea, and greater anticoagulation and antiarrhythmic drug usage. Thus, it would be important for future prospective research in this space to examine whether all AF ablation patients benefit from ICM vs the subset of ablated patients matching these particular characteristics. Additionally, future research should focus on the impact of specific ICM-guided treatment decisions on patient outcomes, and the impact on patient-perspective out-of-pocket costs.

### Limitations

The accuracy and reliability of administrative claims data to identify all healthcare encounters related to the conditions of interest is dependent on the precision and completeness of coding practices, which may vary across providers or institutions.

There may be other important underlying patient characteristics, not available in claims data, that we were unable to account for in the cohort matching process. For example, it was not feasible to match on body mass index, as this was not systematically included in the claims data, being present for only approximately 55% of our population. Additionally, while more granular diagnosis codes for AF type (paroxysmal, persistent, or permanent) became available with ICD-10 codes close to 2016, these were not available for the first several years of our sample selection period, limiting our ability to match on the specific type of AF across our population. A separate analysis by these authors on the utilization of the newer ICD-10 codes showed a markedly slow and inconsistent uptake after the launch of the new system, with 40% of inpatient AF claims in calendar years 2016–2017 still using only the generic code for Unspecified AF (as yet unpublished; data on file). As uptake of the coding improves, future claims-based research will be able to take advantage of the more granular reporting of AF types in the future, although questions still remain about the ability of assessing AF burden based on symptoms alone, in the absence of continuous cardiac data.^25^ The inability to match on AF type in our study could be a potential source of bias if, for example, ICM patients are more likely to be paroxysmal AF patients who may be expected to incur superior outcomes. However, we attempted to mitigate this potential issue by matching on total medical and pharmacy costs and use of individual AF-related medications in baseline, in addition to the other comorbidities.

This analysis was underpowered to examine differences in the rates of ischemic stroke/TIA or major bleeding events, owing to limitations on available patient sample size and low rate of these events. Additionally, data on mortality are undercounted as such data are not consistently available within the Medicare administrative claims dataset, and this analysis did not include linkage to the national Social Security Administration death master file.

Patients who receive any therapeutic CIED devices with an atrial lead capable of continuous atrial monitoring (pacemakers, implantable cardioverter-defibrillators, or cardiac resynchronization therapy) at any time during baseline or follow-up were excluded from the analysis, in order to isolate the impact of long-term continuous AF monitoring with ICMs compared with no continuous AF monitoring. These criteria were applied to both patient cohorts (ICM and non-ICM) to limit bias; however, it is important to note that this exclusion may limit the applicability of the study results in the patients who go on to receive CIEDs after detection of additional arrhythmias. Finally, the generalizability of our findings to the wider population of all AF ablation patients vs the subset of ablation patients matching pre-existing indications for ICM is unknown and would be of interest to study in future prospective research.

## Conclusion

Using data from 2 large US administrative claims datasets, we found that AF ablation patients with long-term ICM monitoring experienced fewer inpatient hospitalizations and total severe cardiovascular events but a greater frequency of routine office-based visits, compared to a matched cohort of patients without ICM. Additionally, ICM patients were more likely to have a change in therapy, such as a repeat AF ablation or discontinuation of OAC or antiarrhythmic medication, during postablation follow-up. These data support the use of ICMs to monitor AF burden and support ongoing therapeutic management in an AF ablation population.
