# Supplementary material for: Healthcare utilization and clinical outcomes after ablation of atrial fibrillation in patients with and without insertable cardiac monitoring
Source: Heart Rhythm O2. 2022 Jan 7;3(1):79–90. doi: 10.1016/j.hroo.2021.12.005 (PMC8859784; doi:10.1016/j.hroo.2021.12.005)
Supplement: Supplemental Tables [file mmc1.docx]

SUPPLEMENTAL TABLES

**Supplementary Table 1. Procedure Codes**

| **Description** | **Current Procedural Terminology, 4th edition (CPT-4) outpatient procedure codes** | **ICD-9-PCS Inpatient procedure codes** | **ICD-10-PCS Inpatient procedure codes** |
| --- | --- | --- | --- |
| Atrial ablation | 93651, 93655, 93656, 93657 | 37.34 | 02583ZZ |
| Atrioventricular (AV) node ablation | 93650 | N/A | 02583ZZ |
| Left atrial appendage (LAA) closure | 33340 | N/A | 02L73DK |
| Holter monitoring or other monitoring for arrhythmia | 93224, 93225, 93229, 93268, 93270, 0295T, 0296T | 89.50, 89.54 | 4A12X45, 4A12X4Z |
| Electrical cardioversion | 92960, 92961 | 37.91, 99.61, 99.62 | 4B02XSZ, 4B02XTZ, 4B09XSZ, 5A2204Z |
| Insertable cardiac monitor – insertion | 33282, 33285 | 37.79 | 0JH602Z, 0JH632Z |
| Insertable cardiac monitor – monitoring | 93285, 93291, 93298, 93299 | N/A | N/A |
| Pacemaker | 33206, 33207, 33208, 33274, 33216, 33217, 33212, 33213, 33221, 33214, 33210, 33211, 33227, 33228, 33275, 33233, 33234, 33235, 33215, 33218, 33220, 33222, 33202, 33203, 33236, 33237 | 0.50, 0.52, 0.53, 0.54, 34.85, 37.70, 37.71, 37.72, 37.73, 37.74, 37.76, 37.77, 37.80, 37.81, 37.82, 37.83, 37.85, 37.86, 37.87, 37.89, 37.94, 37.97, 37.98, 37.99 | 0JH606Z, 0JH605Z, 0JH604Z, 02HK3NZ, 02H63JZ, 02HK3JZ, 02PA3MZ, 0JPT0PZ, 0JWT0PZ |
| Cardiac resynchronization therapy (CRT) device | 33249, 33208, 33207, 33225, 33263, 33264, 33233, 33234, 33235, 33227, 33228, 33229, 33241, 33262, 33263, 33264, 33244 | 0.50, 0.51, 0.52, 0.53, 0.54, 37.70 | 0JH607Z, 0JH609Z, 02H43JZ, 02H43KZ |
| Implantable cardioverter defibrillator (ICD) | 33215, 33216, 33217, 33218, 33271, 33230, 33231, 33240, 33249, 33262, 33263, 33264, 33241, 33244, 33220, 33223 | 0.52, 37.94, 37.96, 37.98 | 0JH608Z, 0JH638Z, 0JH808Z, 0JH838Z, 02H63KZ, 02HK3KZ, 0JH60PZ, 02HN0KZ, 02PA0MZ, 02WA0MZ, 02WA3MZ, 0JWT0PZ, 0JWT3PZ, 0JPT0PZ, 0JPT3PZ, 02PA3MZ |
| Blood transfusion | 36430 | HCPCS Codes: P9010, P9038, P9039, P9040, P9057, P9058, P9070, P9071 | |

**Supplementary Table 2. Diagnosis Codes**

| **Description** | **ICD-9-CM Diagnosis Codes** | **ICD-10-CM Diagnosis Codes** |
| --- | --- | --- |
| Heart failure | 428.1, 428.9, 398.91, 402.01, 402.11, 402.91, 404.01, 404.03, 404.11, 404.13, 404.91, 404.93, 428, 42820, 42821, 42822, 42823, 42830, 42831, 42832, 42833, 42840, 42841, 42842, 42843 | I50.1, I50.8x, I50.9, I09.81, I11.0, I13.0, I13.2, I50.20, I50.21, I50.22, I50.23, I50.30, I50.31, I50.32, I50.33, I50.40, I50.41, I50.42, I50.43 |
| Supraventricular tachycardia | 427.0, 427.1, 427.2 | I47.1, I47.2, I47.9 |
| Wolff-Parkinson-White syndrome | 426.7 | I45.6 |
| Atrial fibrillation | 427.31 | I48.0, I48.1, I48.2, I48.91 |
| All cardiac-related diagnosis codes | 390.x-459.X | I00x-I99.9 |
| Obstructive sleep apnea | 327.23 | G47.33 |
| Systemic embolism | 444.01, 444.09, 444.1, 444.21, 444.22, 444.81, 444.89, 444.9 | I74.01, I74.09, I74.10, I74.11, I74.19, I74.2, I74.3, I74.4, I74.5, I74.8, I74.9 |
| Ischemic stroke/Transient ischemic attack | 435.x, 433.01, 433.11, 433.21, 433.31, 433.81, 433.91, 434.01, 434.11, 434.91, 436.x, 997.02 | G45.x, I63.x, I64, I97.810, I97.811, I97.820, I97.821 |
| Hemorrhagic stroke | 430, 431, 432, 432.1, 432.9 | I60.00, I60.01, I60.02, I60.10, I60.11, I60.12, I60.2, I60.20, I60.21, I60.22, I60.30, I60.31, I60.32, I60.4, I60.50, I60.51, I60.52, I60.6, I60.7, I60.8, I60.9, I61.0, I61.1, I61.2, I61.3, I61.4, I61.5, I61.6, I61.8, I61.9, I62.00, I62.01, I62.02, I62.03, I62.1, I62.9 |
| Major gastrointestinal (GI) bleed | 578.9, 455.2, 455.5, 455.8, 456, 456.2, 530.21, 530.7, 530.82, K57.21, K57.31, K57.33, K57.41, K57.51 , K57.53 , K57.81, K57.91 , K57.93 , K29.01, K29.21, K29.31, K29.41, K29.51, K29.61, K29.71, K29.81, K29.91, K25.0, K25.2, K25.4, 535.51, 535.61, 535.71, 537.83, 562.02, 562.03, 562.12, 562.13, 568.81, 569.3, 569.85, 589.86, 578, 578.1 | K92.2, K29.01, K29.31, K29.51, K29.61, K29.71, K62.5, K31.811, K57.01, K57.21, K57.31, K57.33, K57.41, K57.51 , K57.53 , K57.81, K57.91 , K57.93 , K29.01, K29.21, K29.31, K29.41, K29.51, K29.61, K29.71, K29.81, K29.91, K25.0, K25.2, K25.4, K25.6, K26.0, K26.2, K26.4, K26.6, K27.0, K27.2, K27.4, K27.6, K28.0, K28.2, K28.4, K28.6, K92.1 |
| Major brain bleed | 852.0, 852.1, 852.2, 852.3, I60.22, I60.30, I60.31, I60.32, I60.4, I60.50, I60.51, I60.52, I60.6, I60.7, I60.8, I60.9, I61.0, I61.1, I61.2, I61.3, I61.4, I61.5, I61.6, I61.8, I61.9 | S06.4xxx, S06.5xxx, S06.6xxx |
| Other major bleed | 362.81, 363.61, 376.32, 423, 568.81 | H05.233, H31.309, H35.60, H35.61, H35.62, H35.63, I31.2, K66.1 |
| Minor bleed  *Note: Any bleed with evidence of transfusion were classified as a Major bleed* | 596.7, 599.7, 623.8, 626.2, 626.6, 626.8, 719.1, 784.7, 784.8, 786.3, 459, 285.1 | R31.9, N92.1, N93.9, M25.00, R04.0, R04.1, R04.2, R04.89, R04.9, R58, D50.0, N02.9 |
| Ischemic heart disease | 410.x, 411.x, 412, 413.x | I20-I25 |
| Hypertension | 401.1, 401.9, 401.0, 402.00, 402.01, 402.10, 402.11, 402.90, 402.91, 403.10, 403.11, 403.90, 403.91, 404.00, 404.01, 404.02, 404.03, 404.10, 404.11, 404.12, 404.13, 404.90, 404.91, 404.92, 404.93, 405.01, 405.09, 405.11, 405.19, 405.91, 405.99, 437.2 | I10, I11.0, I11.9, I12.0, I12.9, I13.0, I131.0, I131.1, I13.2, I15.0, I15.1, I15.2, I15.8, I15.9, I16.0, I16.1, I16.9, N26.2, I67.4 |
| Dementia | 290.0, 290.3, 290.42, 290.41, 290.4, 290.43, 290.11, 290.1, 290.13, 290.12, 290.21, 290.2, 331.82, 331.11-331.19, 292.82, 291.2, 294.21, 294.2, 294.11, 294.1 | F01.5, F01.51, F01.50, F03, F03.9, F03.91, F03.90, G31.83, G31.0, G31.09, F02.8, F02.81, F02.80, F10.27, F10.97, F18.97, F18.17, F18.27, F13.27, F13.97, F19.97, F19.17, F19.27 |
| Diabetes | 250.0x, 250.1x, 250.2x, 250.3x, 250.4x, 250.5x, 250.6x, 250.7x, 250.8x, 250.9x, | E10.x, E11.x |
| Myocardial infarction (MI) | 410.0x , 410.1x, 410.2x, 410.3x, 410.4x, 410.5x, 410.6x, 410.7x, 410.8x, 410.9x, 412 | I21.01 , I21.02, I21.09, I21.11, I21.19, I21.21, I21.29, I21.3, I21.4, I22.0 , I22.1, I22.2, I22.8, I22.9, I25.2, I21.A1, I21.A9, I21.9 |
